# Graphical study of reasons for engagement in physical activity in European Union

**DOI:** 10.1186/2193-1801-2-488

**Published:** 2013-09-26

**Authors:** Daniel Ríos, Marta Cubedo, Martín Ríos

**Affiliations:** University of Barcelona, Avda. Diagonal 643, 08028 Barcelona, Spain

**Keywords:** Physical activity, Health, Europe, Motivational factors, Principal component analysis, Cluster analysis

## Abstract

We collect data on 15 reasons why people in the 27 EU countries engage in physical activity, from the European Commission’s Special Eurobarometer. A graphical output was obtained using classical Principal Component Analysis techniques in order to analyse types of motivation in the EU. Cluster Analysis method were used to define the interrelationship between the data in the 27 countries. People in Sweden, Denmark and Finland were the most highly motivated. High rates were detected in Austria, Germany, Slovenia, Estonia, Luxembourg and Latvia while low rates were found in Bulgaria, Romania, Czech Republic, Greece, Spain, Hungary, Italy, Lithuania, Poland, Portugal and Slovakia. The lowest motivation rates were in the Netherlands. Regarding the reasons for engaging in exercise (a sport or physical activity), we observed two motivation types. The first group was related to health and physical appearance while the second was associated with social reasons: to be with friends, to better integrate into society, to meet people from other cultures. For citizens of Latvia, Bulgaria and Romania, health and physical appearance carried greater importance than the European average while for citizens of Germany, Finland and Sweden the second motivation type was higher than the European average.

## Introduction

Physical activity is closely related to reduced morbidity and mortality in developed countries, where rates of non-communicable diseases have increased rapidly in recent years, (World Health Organization 2002). The World Health Organization, (Bauman et al. 2005), recommended that people engage in physically active daily.

In spite of the benefits of physical activity, most people in the European Union (E. U.) do not exercise, (defined as the deliberate performance of a physical activity that requires exertion). Over 30% report that they seldom or never engage in any physical activity and 25% are virtually inactive (European Commission, 2010). Research suggests that up to 50% of people who start an exercise programme drop out within the first six months, (Wilson & Brookfield, 2009). Therefore, it is of interest to study what motivates people to exercise. This information would be of useful to design strategies to encourage people to participate in and adherence to exercise programmes. Previous studies have suggested that a better understanding of motivation would benefit initiatives to promote physical activity (Dishman & Sallis, 1994; Trost et al. 2002) reviewed the range of demographic, biological, psychological, social, cultural and physical environment factors associated with physical activity in adults.

Motivation to exercise differs between stages of live (Allender et al. 2006). (children and adolescents, adults and elderly) and countries. It is not appropriate to base physical activity recommendations for children and adolescents on evidence of morbidity and mortality; however in this group proposing physical activity as a way to enhance social interaction would be suitable. During adulthood, there is a need to reduce premature risk factors for a range of chronic diseases and conditions and to maintain an energy balance. Furthermore physical activity has the potential to improve mental health. In the elderly motivation to engage in physical activity may be that it slows the ageing process and contributes to maintaining functional capacity.

Early work on motivation for sport and exercise (Alderman & Wood, 1976) proposed that young athletes in Canada participated in sports for fitness, challenge and fun. (Williams & Cox, 2003) stated that the reasons for participation were linked to social prestige and status. Other reasons for engaging in these practices include personal challenge and satisfaction, fitness and the desire to lose weight and improve health, (Daley & Duda, 2006; Edmunds et al. 2006). The role of appearance/weight, health/fitness and social engagement motives in exercise participation was tested by (Markland & Ingledew, 2007; Ingledew & Markland, 2008).

A theoretical approach to determining the motivation behind participation in exercise and physical activity can be found in (González-Cutre et al. 2010).

In the present study, we explored the reasons why people in 27 European countries exercise and we propose strategies for practical application of these data in the E. U. We used principal component analysis (PCA) (Anderson, 1958; Muirhead 1982), to display the results according to the different populations. Small distances on the graph indicate similar populations, whereas large distances show dissimilar populations in their motivation to exercise. This analysis allowed us to identify motivational level differences between countries. For this purpose, 15 variables corresponding to types of motivation to engage in sport or physical activity were considered for each country. First, we classified the countries using cluster methods. We then plotted the output data for these countries as points on a graph in a low-dimensional Euclidean space. Classical PCA was used to obtain the final graphical output.

## Methods

### Reported cases

The data presented in this study were provided by the European Commission (2010) and can be further filtered, tabulated, charted and downloaded. They are available at: http://ec.europa.eu/public_opinion/archives/ebs/ebs_334_en.pdf. This information permit to repeat the work.

The data analysed correspond to 27 European countries and one “average” country. The following 15 variables were proposed as reasons to practice sport or engage in physical activity: to improve health, to improve fitness, to relax, to have fun, to improve physical appearance, to improve physical performance, to control weight, to be with friends, to counteract the effects of ageing, to improve self-esteem, to develop new skills, for the spirit of competition, to make new acquaintances, to better integrate into society and to meet people from other cultures.

### Statistical analysis

As the data (proportion of citizens in each country), were obtained from large populations they can be considered as random normally distributed variables.

Each country and one “average” country was associated with the *n-*dimensional random vector *X*_*h*_ *= (X*_*h1*_*,…,X*_*hn*_*)* where *X*_*hi*_ represents the proportion of citizens in the country *h* that have the motivation *i = 1,…,n,* to participate in sport or engage in physical activity (see “Reported cases” section above). In this study *h = 27 + 1 = 28* and *n = 15.*

For each country, we have a realization *x = (x*^*1*^*,…,x*^*n*^*).* The distance between the population *x = (x*^*1*^*,…,x*^*n*^*)* and *y = (y*^*1*^*,…,y*^*n*^*)* is given by .

The interdistance matrix *D* was used to obtain a graphical output through taxonomic analysis. Similar objects were grouped into clusters (subsets of a set of objects); two clusters may be separate. On the basis of the distance between pairs of objects, the distance between the new cluster and all other objects was defined. We used the unweighted-pair group method with arithmetic mean (UPGMA).

PCA was performed using the distances obtained. This method represents a set of points in *n-*dimensional space by a subspace of smaller dimensions. The main purpose is to reduce the data from a large number of variables to a smaller number of components, there by facilitating the detection of similarities and differences between the countries.

Statistical significance was set at *p < .05.* All analyses were conducted using the (SPSS Statistical Package version 17.0 2009; STATGRAPHICS® Centurion XVI User Manual Statgraphics® Centurion, STATGRAPHICS® Centurion XVI User Manual STATGRAPHICS® Centurion XVI User Manual 2010).

## Results

**Figure 1:**
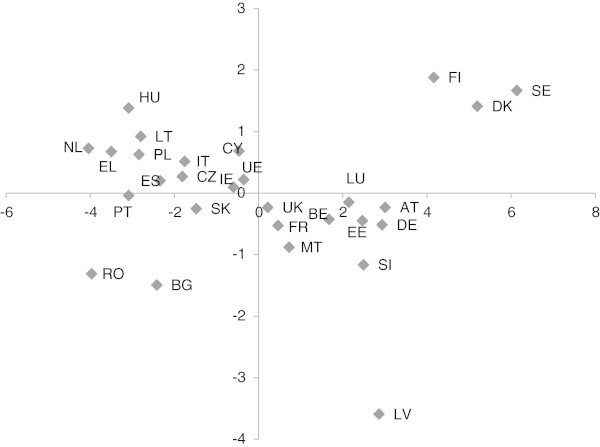
**Two-dimensional graphical display of 27 European countries and one average country, based on the PCA.** The two dimensions account for 69,3% of the inertia (BE: Belgium, BG: Bulgaria, CZ: Czech Republic, DK: Denmark, DE: Germany EE: Estonia, EL: Greece, ES: Spain, FR: France, IE: Ireland, IT: Italy, CY: Republic of Cyprus, LT: Lithuania, LV: Latvia, LU: Luxembourg, HU: Hungary, MT: Malta, NL: The Netherlands, AT: Austria, PL: Poland, PT: Portugal, RO: Romania, SI: Slovenia, SK: Slovakia, FI: Finland, SE: Sweden, UK: The United Kingdom, UE: Average country).

The PCA is summarised in Table 1. All coefficients of the principal components and all eigenvalues are shown. The inertia percentages accounted for by the first two axes were 60.4% and 8.9%. These components explained more than 69% of the total variation. The first component was the “size”, with all coefficients positive, and it represents the overall motivation. In the second component, the coefficients corresponding to improving health, increasing fitness, having fun, looking better, boosting physical performance, controlling weight and counteracting the effects of ageing were negative, while the rest (to relaxing, being with friends, improving self-esteem, developing new skills, exercising the spirit of competition, making new acquaintances, better integrating into society, meeting people from other cultures) were positive. These observations highlight two types of motivation to engage in sport or physical activity, the first related to health and physical appearance and the second associated with social reasons. This component takes large values for countries where social reasons are more important and small values for countries where reasons related to health and physical appearance are more important. The two-dimensional display obtained is shown in Figure 1.

**Table 1:** **Eigenvalues and eigenvectors of the correlation matrix for 15 measurements of percentages of the reasons for exercising in 27 European countries and one average country**

Reasons for exercising	First	Second
Eigenvalue	8,45112	1,23908
Variance that it account	60.365	8,851
Improve health	0.225355	−0.388504
Improve fitness	0.26383	−0.30367
Relax	0.217934	0.0302201
Have fun	0.195627	−0.110219
Improve physical appearance	0.222883	−0.094510
Improve physical performance	0.220278	−0.24821
Control weight	0.284559	−0.192121
Be with friends	0.280888	0.119302
Counteract the effects of ageing	0.262984	−0.045768
Improve self-esteem	0.303271	0.0434798
Develop new skills	0.296846	0.0935058
Spirit of competition	0.308349	0.0666423
Make new acquaintances	0.313184	0.202801
Better integrate into society	0.12407	0.693219
Meet people from other cultures	0.279221	0.288538

The first PCA component was positive (indicating globally higher motivation to exercise) in 8 countries (Sweden, Germany, Finland, Austria, Germany, Estonia, Luxembourg, Slovenia and Latvia; from highest to lowest) and negative (indicating a globally lower motivation to exercise) in 12 countries (the Netherlands, Bulgaria, Romania, the Czech Republic, Greece, Spain, Hungary, Italy, Lithuania, Poland, Portugal and Slovakia; from highest to lowest). The second PCA component was positive (indicating greater importance of social motives to exercise) in 3 countries (Germany, Finland and Sweden; from highest to lowest) and negative (indicating higher motivation to exercise related to health and physical appearance) in 3 countries (Latvia, Bulgaria and Romania; from highest to lowest).

**Figure 2:**
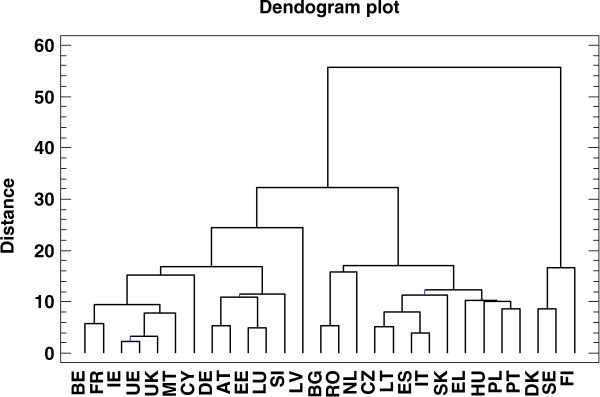
**Cluster analysis using UPGMA method and classification of groups by to engage in sport and physical activity: Group I: DK: Denmark, FI: Finland and SE: Sweden, Group II: AT: Austria, DE: Germany, EE: Estonia, LU: Luxembourg and SI: Slovenia.** Group III: LV: Latvia. Group IV: BE: Belgium, CY: Republic of Cyprus, FR: France, IE: Ireland, MT: Malta, UK: The United Kingdom and UE: the “average” country. Group V: CZ: The Czech Republic, EL: Greece, ES: Spain, HU Hungary, IT Italy, LT: Lithuania, PL: Poland, PT: Portugal and SK: Slovakia. Group VI: NL: The Netherlands. Group VII: BG: Bulgaria and RO: Romania.

On the basis of the tree reconstructed by UPGMA (Figure 2) and Figure 1, we identified seven population groups:

- Group I: Denmark, Finland and Sweden.
- Group II: Austria, Germany, Estonia, Luxembourg and Slovenia.
- Group III: Latvia.
- Group IV: Belgium, Republic of Cyprus, France, Ireland, Malta, The United Kingdom and the “average” country.
- Group V: The Czech Republic, Greece, Spain, Hungary, Italy, Lithuania, Poland, Portugal and Slovakia.
- Group VI: The Netherlands.
- Group VII: Bulgaria and Romania.

Groups I and II were the most motivated to exercise (Figure 2). Countries included in Groups VI and VII had the lowest values for the first component, thereby indicating the lowest motivation to engage in physical activity. The countries with near-average values for the first component are in Group IV, suggesting that these countries were in the mid-range.

For the second component, the largest values corresponded to Group I and the lowest to Group III while moderately high values corresponded to Group V. Group IV countries showed high motivation although the second component was close to zero.

## Discussion

Here we examined the reasons why citizens in the E. U. engage in sport and physical activity. A graphical representation of the percentage of the populations with a specific motive to exercise was used to plot the data for the various countries. This approach was useful because all coefficients of the first component clearly represented the “size”, i.e., the overall reasons for engaging in sport or physical activity. The second axis had 7 negative and 8 positive components. This axis represented the type of motivation, i.e. the populations of countries represented by points whose second component had a high positive value were considered to be highly motivated by health and physical appearance. Conversely when the coordinates of the points had a second component with a lower this was interpreted as indicating population highly motivated to engage in sport and physical activity for social reasons. The taxonomic method was used to group the countries.

Our findings reveal that the types of motivation vary between E. U. countries. On the basis of the tree and principal component representation, the Nordic countries: Denmark, Finland and Sweden showed the largest proportion of citizens motivated to engage in sport or physical activity and this motivation was social. Latvia showed high motivation reasons being related mainly to health and physical appearance. In contrast the Netherlands showed the lowest motivated population and the driving reasons to engage in exercise were mainly of a social nature. A low proportion of the people in Bulgaria and Romania were motivated for exercise and those that did engage in this activity were driven by reasons mainly associated with health and physical appearance.

The countries comprising Group I (Nordic countries) were found to be the most physically active in the E. U. Less than 20% of the population of these countries report never exercising or engaging in sport (European Commission 2010). This finding is explained by the fact that these countries are the most motivated with regard to exercise. Group V and Group VII counties are reported to have the fewest citizens engaging in sport or exercise, as confirmed by these countries showing the lowest motivation to exercise. It therefore follows that countries with high proportion of motivated citizens engage in more physical activity and sport than the “average” country and those whose populations show little interest in physical activity are less active than “average” country.

The countries in Groups II and IV were highly motivated to exercise and were driven mainly by reasons of health and physical appearance. In Figure 1 these countries are located in quadrant IV, having the following coordinates; positive abscissa and negative ordinate. A greater proportion of their populations engaged in sport and physical activity than the “average” country.

Group V countries showed low motivation to exercise however, those exercising reported motivation linked to social interactions. In Figure 1 these countries are located in quadrant II, having the following coordinates; negative abscissa and positive ordinate. A lower proportion of their populations engaged in sport and physical activity lower than the “average” country.

On the basis of all these observations, we can deduce that the motivations related to health and physical appearance is more decisive for physical activity. However, there are some exceptions.

In the Netherlands a low proportion of the population was motivated to engage in sport and physical activity however, curiously, this population is among the most physically active as only 28% report never engaging in sport or physical activity. People (73%) in the Netherlands expressed satisfaction with local councils regarding sports and exercise provision and report a high degree of satisfaction with their local sports clubs, which are particularly popular.

The people in Latvia were found to be highly motivated however, a proportion of citizens, more than average of the E. U. reported never taking any exercise. This finding could be attributed to the poor economic situation of the country and consequently of its people.

Bulgaria and Romania are the two countries that most recently joined the E. U. People in these countries show little inclination to engage in sport and physical activity and motivation was essentially related to health and the regulation of energy expenditure. A relatively high proportion of citizens, more than average of E. U. reported never engaging in physical exercise. This finding is explained by the lack of opportunities to be physically active and to socio-economical limitations.

## Conclusions

It is widely accepted in the E. U. that sport and physical activity contribute to health. We show that in most E. U. countries there is a link between health and the motivation to keep physically fit and active. Exceptionally, in the Nordic countries: Denmark, Finland and Sweden most people have a strong social motivations to exercise and a high proportion of the people in these countries do more physical exercise than the E. U. average.

People in the Netherlands lack motivation to exercise and when they do engage in this activity it is essentially for social reasons. Therefore its inhabitants engage in less sport and physical activity than the E. U. average.

Bulgaria and Romania show low motivation. People in these countries report being motivated by health issues and are physically more inactive than E. U. average.

One of the main objectives of the article; to present a large number of data in summary form without sacrificing too much information, has been achieved and also aims to encourage future researchers to present information accompanied by methods of summary representation.

Others detailed socio-demographic information to help understand the reasons why sections of European society (gender, age, education levels, ..), engage in sport and physical activity. But this should be analyzed by each country, and is outside the scope of this study.
